# Membership, Neighborhood Social Identification, Well-Being, and Health for the Elderly in Chile

**DOI:** 10.3389/fpsyg.2020.608482

**Published:** 2021-01-18

**Authors:** Emilio Moyano-Díaz, Rodolfo Mendoza-Llanos

**Affiliations:** ^1^Faculty of Psychology, University of Talca, Talca, Chile; ^2^Department of Social Science, School of Psychology, University of Bío-Bío, Chillán, Chile

**Keywords:** well-being, neighborhood councils, social identification, older adults, belong to social organizations

## Abstract

The world’s elderly population is growing, and in Chile they represent 16.2% of the total population. In Chile, old age is marked by retirement, with a dramatic decrease in income that brings precariousness. Older adults are economically, socially, and psychologically vulnerable populations. This condition increases their likelihood of disengaging from their usual social environment, facilitating their isolation, sadness, and discomfort. From the perspective of social identity, well-being (WB) can be explained by two principles: social groups’ importance for health and people’s psychological identification with those groups. This study analyzes the relationships between belonging to the neighborhood and extra-neighborhood groups and neighborhood social identification with WB. Urban or rural location and gender are measured, and the sample is 1,475 older Chilean adults of both sexes. The results show that the majority are not members of social groups (52%), and the remaining 48% are members of one or two groups or organizations (42.65%). Only 4.47% belong to three groups or organizations. Those who belong to groups obtain higher scores, emotional–mental WB, and positive emotions than older adults who do not belong to any organization. Urban and rural older adults have the same level of WB. Membership in close social organizations (neighborhood councils) or distant ones (clubs for the elderly and religious groups) causes different WB associations. Membership in neighborhood councils reduces gender differences in self-assessment of health. This result supports the idea that participation in heterogeneous groups with a shared sense of belonging to the neighborhood is associated with higher WB and lower perceived loneliness. Social identification with the neighborhood, rather than belonging to the group, had the most widespread impact on WB and health indicators. The variable social identification with the neighborhood was consistently associated with indicators of hedonic WB.

## Introduction

In the United Nations (2015) reported that almost 901 million people were over 60, comprising 12% of the global population. An aging society is a significant issue in many countries. However, in developed countries, a large, growing elderly population has occurred over a long period. In contrast, low- or middle-class Latin American countries have seen a faster and more drastic increase.

The world’s population of older adults is growing. In the Southern Cone countries, Argentina, Uruguay, and mostly Chile have experienced accelerated aging of their population and in a context of inequality (Fuentes-García et al., 2013). In Chile, older adults represent 16.2% of the population, and in 30 years, one in four people will be over 60 (Instituto Nacional de Estadísticas, 2017). Thus, the decrease in the birth rate and the sustained economic growth in Chile brought a significant increase in life expectancy at birth, which has tripled in the last century. In 1900, life expectancy was at 23.6 years for women and 23.5 years for men, and today, for the period 2015–2020, it is 82.1 for women and 77.3 for men.

Therefore, the challenge is not only to live longer but also to live well, with the best quality of life and well-being (WB) possible. However, older adulthood in Chile is marked by retirement with a dramatic decrease in income, bringing precariousness. Thus, older adults are economically, socially, and psychologically vulnerable populations (Grenier et al., 2017; Romaioli and Contarello, 2019). His or her change of role—from worker to pensioner—is also accompanied by a change in his or her environment. He or she will no longer go to the usual workplace, producing an ecological transition that is both a consequence and an instigator of development processes (Bronfenbrenner, 1987, p. 46). This vulnerable position increases the likelihood that older adults disengage from their usual social environment, facilitating their isolation, sadness, and discomfort. Thus, belonging to social groups can counteract this vulnerable position by providing the possibility of social interaction, participation, and social support.

Social activity is considered part of an active lifestyle, alongside physical, and cognitive activities. Social activity has been demonstrated to be beneficial for various health outcomes, including physical health status, mental health status, and quality of life (James et al., 2011; Stav et al., 2012; Lee and Kim, 2014). Numerous studies indicate that social participation positively affects older adults’ health (Tomioka et al., 2015; Su et al., 2018; Tomioka et al., 2018).

According to the World Health Organization (World Health Organization (WHO), 2002), social activities are an essential component of “active aging,” and the Active Aging Framework has been adapted as a global strategy in aging policies, practices, and research over the last decade (Narushima et al., 2018). Older adults are at an elevated risk of adverse health effects associated with social isolation and loneliness. Social participation is considered a modifiable determinant of health and WB has been proposed to reduce this risk. However, there is limited knowledge to date about the patterns of social activities among older adults (Dawson-Townsend, 2019).

The neighborhood environment and its relationship with group membership, social participation, and the health of the elderly is one of the relevant issues of environmental gerontology (Kendig, 2003; Moore, 2014). Zheng et al. (2019) assert that the fundamental goal of building “age-friendly communities” (Menec et al., 2011) is to help the elderly access more opportunities for participation and better health, but little is known about the complex relationships between neighborhood environment and elderly health. It has been observed that older adults who perceive that the neighborhood gives the possibility of being healthy experience WB (Sánchez et al., 2017). Neighborhood social reciprocity is an essential aspect of neighborhood social capital, and many previous studies have found that it benefits people’s mental health status (Adjayegbewonyo et al., 2018; Wang et al., 2019; Xiao et al., 2019). Wang et al. (2019) found that neighborhood social reciprocity influenced Chinese seniors’ mental health directly through the frequency of physical activity, social interaction with neighbors, and volunteering experience. The perception of insecurity in the neighborhood has a significant effect on depressive symptoms; however, a greater sense of belonging to the community dampened or had a significant mediating effect on this relationship (Gonyea et al., 2017) and, more generally, on mental health (Fong et al., 2019). Kim and Kawachi (2017) found that neighborhoods with the right interpersonal environment could make it easier for older adults to participate more in social interactions and exchange information useful for their health.

A set of concepts similar to social capital, such as social engagement, social involvement, social networks, social integration, and social gatherings, has been used interchangeably with social participation. Thus, social participation appears as a polysemy concept, which includes membership in social or community clubs or organizations or activities such as individual activities (e.g., hobbies, neighborhood relations) and community activities (e.g., local events, volunteers, senior centers, and religious; Levasseur et al., 2010). Besides, a systematic review of social isolation interventions suggests that a social activity in-group format was more likely to be beneficial than a one-to-one format (Dickens et al., 2011).

Social identity theorists have proposed that people’s sense of identity is also derived from their membership in social groups (Tafjel, 1978; Turner et al., 1994; Fong et al., 2019). They distinguish between social identities as a cognitive psychological aspect and social identification as an affective psychological component. On one hand, we analyze and count membership of groups or organizations understood as a central part of social identity, and on the other hand, we understand social identification with the neighborhood as people’s strength or psychological closeness. Thus, people have various social identities, corresponding or eventual social identifications, where the former are a precondition for developing the latter (Zacher et al., 2019). The social identity approach suggests that membership and social identification with important groups or organizations are primary determinants of WB and health (Fong et al., 2019). Thus, the non-membership of social groups and loneliness in older adults are most likely associated with worse health and lower WB. “Group life is central, a key source of meaning, purpose, and direction.” “the groups make life worth living, and they are what we live for” (Haslam et al., 2018, p. 17). Groups can also be a source of discomfort, so a complementary hypothesis from the approach of the theory of social identity is that a person will generally experience health-related benefits or costs of a given group membership only to the extent that they identify with the group. In this study, membership in neighborhood groups and extra-neighborhood groups will be analyzed, as well as their neighborhood social identification (NSI) and their relationships with WB and health.

It is possible to establish the determinants of WB and health associated with social membership and participation at different levels of analysis: territorial (e.g., urban–rural), individual (sociodemographical variables as sex and educational level), and neighborhood or extra-neighborhood level. These will have different impacts on the WB of people, and they interact with each other. Regarding territorial determinants, some studies have shown that Chinese older people in rural regions, compared with their urban counterparts, have a lower perception of satisfaction with life and happiness (Li et al., 2015). Overall, however, the lack of services, including local health care facilities, was less important than the attachment to place and social capital associated with aging in place. So, many rural-dwelling older adults reported that the positive aspects of rural residence, such as attachment to community and familiarity, create a sense of belonging that far outweighs the negative (Carver et al., 2018).

The relationships between social participation and health varied according to social activity and the rural–urban context. The rural dwellers were less socially active than their urban counterparts, and in the rural areas, religion and the arts were associated with later health perception (Vogelsang, 2016). Pan (2018) examined the relationship between social capital in the form of cognitive (trust, family support) and structural aspects (social membership, activity frequency) and the life satisfaction of the Chinese elderly in a rural area. Trust and family support were positively associated with life satisfaction, whereas social membership was negatively associated with life satisfaction. Activity frequency did not show any significance in relationship with life satisfaction.

Tomioka et al. (2018) suggest that participation in various social groups effectively maintains older people’s effectance, while the beneficial effect of each type of social participation on effectance is more substantial for females than for males. Furthermore, the beneficial effects of frequent participation may be stronger for women than for men. Another study (Zaitsu et al., 2018) reported that Japanese participants involved in social groups with greater diversity had better self-rated health than people who did not participate in social groups. Participation in gender-diverse groups was associated with the best health profile.

Amagasa et al. (2017) indicate that frequent participation at church was related to the low prevalence of depressive symptoms in older women than in older men. Amagasa et al. (2017) have sustained that higher community involvement in older women living with others was also associated with a lower risk of psychological distress. Community involvement provides older women with mental health benefits regardless of the individual relationship level, so promoting community involvement may be an effective strategy for healthy mental aging. Tomioka et al. (2017) examined the cross-sectional associations of the type, frequency, and autonomy for social participation with physical and mental health. Overall, positive associations of the frequency and autonomy of social participation were stronger in females than males. They concluded that obligatory social participation had significantly lower effects on mental health than voluntary participation and occasionally non-participation; there is a possibility that obligatory social participation has harmful influences on the mental health of the community-dwelling elderly.

Preceding research has suggested that participation in social activities, such as neighborhood, retired, senior, or charitable associations, alleviates depressive symptoms (Chiao et al., 2011; Cruwys et al., 2013). Participation in physical, social, and religious activities was associated with a decreased risk of depression in the elderly. The risk of depression was much lower in the elderly who participated in two or three of the abovementioned types of activity than that in the elderly who did not (Roh et al., 2015). The protective effects of social activity involve various psychosocial mechanisms, including increased social support and buffering distress (Teo et al., 2013). Regular participation in leisure-time physical activity has many benefits, including postponing premature mortality (Janssen et al., 2013; Lahti et al., 2014; Haak et al., 2019), reducing the development of chronic non-communicable diseases (Annear et al., 2009; Deplanque et al., 2012), and improving quality of life (Sánchez-Villegas et al., 2016).

A positive social environment and opportunities for social participation have demonstrated associations with positive health outcomes for older adults in North America (Glass et al., 1999; Gilmour, 2012), East Asia (Hsu, 2007; Kondo et al., 2007; Gao et al., 2018), and Europe (Bennett, 2005; Sirven and Debrand, 2008; Vogelsang, 2016). However, the literature on the subject in Latin America is very scarce, and we do not know if the same type of results would be obtained here. Simões Oliveira et al. (2016) report that older Brazilians who participate in social groups had better quality of life scores in the social and intimate domains. In Chile, a study showed four social participation sources associated with personal satisfaction: the home, the rural environment, social policy, and religiosity. The latter is an essential source of association with greater participation for women than men, although it equals after 80 years old (Herrera Ponce et al., 2014). In another Chilean population sample study, Fernández et al. (2018) identified active aging predictors. In this study, social variables (social and labor participation understood as active involvement in groups or work activity) showed the best predictive capacity, followed by lifestyle variables and individual characteristic variables (Fernández et al., 2018). A comparative study of the relationship between participation in social organizations and life satisfaction in older adults between 2011 and 2013 shows contradictory results. For 2011, the relationship between both variables was positive, while for 2013, it was negative (Alvarado et al., 2016). In general, older adults receive essential health benefits from more robust social capital. Nevertheless, the mechanisms behind these associations are not fully understood (Ho et al., 2018).

### The Present Study

We believe that belonging to the neighborhood and extra-neighborhood groups and social identification with the neighborhood determine the elderly’s WB and health. Membership is understood here as a proxy for social participation (Levasseur et al., 2010), and it will be measured or accounted for. The NSI is a measure of how positive the inhabitants feel their belonging to the place where they live, implying comfort, a sense of welcome and support, collaboration and trust between neighbors and, more broadly, a sense of community. Consequently, this study’s objective is to determine in older adults of both sexes the relationships between their membership to neighborhood and extra-neighborhood groups, NSI, and WB and health.

The global general working hypothesis is that belonging to the neighborhood and extra-neighborhood groups and organizations and social identification with the neighborhood are factors that positively determine the hedonic and health WB of the elderly. However, although social membership and social identification with the neighborhood are associated with each other (Zacher et al., 2019), they are not similarly related to WB and health indicators.

In this work, we consider two hypotheses that are complementary to each other. In the first place, and from the perspective of social identity, the structural hypothesis (Pan, 2018) proposes that the greater the social membership, the greater the indicators of WB and health. From the perspective of the affective component of social participation, we expect that the greater the social identification with the neighborhood in the elderly, the greater the reported WB and health. Concerning the sociodemographic variables, urban people are expected to have higher membership, higher social identification of the neighborhood, and better WB and health than rural people. Regarding sex, we expected women to have higher membership and NSI than men and less WB and worse health.

## Materials and Methods

This study corresponds to secondary data analysis, which is why the approval of an ethics committee associated with this article is not necessary. According to the information available on the Ministry of Health’s website, it is possible to review the instrument used, which allows us to confirm that the study considers delivering a letter of presentation of the study and the informed consent before starting the application of the survey. We can also observe no potential risk for the participants because anonymity was guaranteed throughout the data.

### Participants

This research used information from the Survey on the Quality of Life from the Ministry of Health of Chile, and Department of Epidemiology (2016)^1^, which was applied nationally to a random sample of participants from 15 years of age and older (*N* = 6,099). Of those participants, 1,475 older adults were selected for this study. The participants were older than 65 years (59.93% women) with an average age of 73.42 years and *SD* = 6.36 (95.25% of the sample was ≤85 years old). Regarding the distribution according to educational level, 7.9% of the participants completed university education or superior, 20.9% completed secondary education, 11.3% had incomplete secondary education, and the remaining 59.9% completed primary education or less. A total of 79.05% of the participants were urban inhabitants, and 20.95% were rural inhabitants.

### Measures

The selection of items from the Survey on Quality of Life allowed making an instrument *ad hoc* consisting of four parts.

(1) *Membership* in neighborhood councils and extra-neighborhood groups (church and senior clubs): To measure membership, we count and add the participation of each subject to a neighborhood council, senior citizen club, and church. Therefore, the membership variable has a possible value between zero and three.
(2) *Neighborhood social identification scale*: This was used to measure the degree to which the person perceives belonging to the place or immediate environment they live and integrate. This scale includes a feeling of closeness and comfort, collaboration, support, trust, and loyalty with neighbors. This scale considers 19 items answered in a five-point Likert format, where 1 = strongly agree to 5 = strongly disagree. Examples of items are as follows: “In general I feel very comfortable living in this neighborhood,” “I feel loyalty to the people in my neighborhood,” or “Living in this neighborhood gives me a sense of community” (complete instrument in section “Appendix 1”). According to the principal component analysis carried out, we found a univariate factorial structure of the scale, which explained 46% of the variance. The scale reliability obtained was α = 0.92 and ω = 0.92. We separated between those who have more and less social identification with the neighborhood, using cutoff scores above and below the median, and performed comparisons in WB and health variables. Thus, two groups were formed: high social identification with the neighborhood (score above the median = 3.58) and another with low social identification with the neighborhood (score below the median).
(3) *Health perception* corresponds to a five-point mono-item “In general, you would say that your health is” 1 = “bad” or 5 = “excellent.” This question is part of the questionnaire of Hennessy et al. (1994) measuring health-related quality of life (Moyano Díaz and Ramos, 2007, Spanish version).
(4) *Hedonic* WB: This includes five indicators about emotionality, mental–emotional WB, life satisfaction, and perceived loneliness. The description of each of the measures is provided below.

*Two mono-items to measure satisfaction with life and mental–emotional* WB, respectively: “All in all, how satisfied are you with your life?” Participants answered on a 10-point scale, ranging from “extremely unsatisfied” (1) to “extremely satisfied” (10). This question is the most extensive single-item question designed to measure life satisfaction, and it has shown good psychometric properties (Unanue et al., 2017). “All in all, how satisfied are you with your mental WB or emotional WB?” Participants answered on a seven-point scale, ranging from “very bad” (1) to “very good” (7).

#### Emotionality

To measure emotionality, we use the question “How often have you felt during the last 2 weeks?” This question refers to eight emotions: four positive (optimistic, happy, calm, and determined) and four negatives (angry, worried, sad, and tired). Participants responded on a five-point scale, ranging from “never” (1) to “always” (7). Score corresponds to the calculated means, and reliabilities to positive and negative emotions are α = 0.78 and ω = 0.79 and α = 0.73 and ω = 0.73, respectively.

#### Perceived Loneliness

To measure perceived loneliness, we use three questions: “How often do you feel that you lack company?,” “How often do you feel excluded or left out by others?,” and “How often do you feel isolated by others?” Participants answered on a three-point scale: “rarely” (1), “sometimes” (2), and “almost always” (3). The score corresponds to the calculated means, and their reliabilities were α = 0.86 and ω = 0.87.

### Data Analysis

First, frequency distribution analyses and reliabilities were obtained. To check the normality, this study applied the statistical method of skewness and kurtosis following Kline’s (2011) skewness (*sk* < 3) and kurtosis (*k* < 10) criteria. Hence, the normality distribution was achieved. Second, in the data analysis, only the scales with all the items answered were included and those in which the Cronbach’s α and McDonald’s ω coefficient were higher than 0.70. Then, Pearson’s correlation coefficients were used to calculate the associations between membership, social identification with the neighborhood, perceived economic security, health, and hedonic WB indicators in the entire sample.

The entire sample was divided into study groups according to sociodemographic characteristics: urban–rural distinctions, sex, belonging to territorially close groups (neighborhood council), and distant groups (older adults clubs and religious groups), for the indicators of health and hedonic WB. This separated analysis was performed to explore the independent effect of belonging to a different group on health and hedonic WB indicators. Finally, one comparison was made to evaluate the impact of belonging to the neighborhood council as close territorial organizations related to sex and perceived health and hedonic WB indicators.

To respond to the objectives of the study, Pearson’s correlation analysis and comparison of means were carried out using the Student’s *t* test or ANOVA and its respective effect size calculation using Cohen’s *d*. Additionally, we conducted a 2 × 2 ANOVA to assess whether there is a differentiated impact on neighborhood membership about the sex of the participants. Tukey’s HSD *post hoc* test was used because it is more conservative (minor type I error). To interpret the correlations, Gignac and Szodorai’s (2016) criteria were used. They suggest that the interpretation of social studies’ correlations would correspond to small, medium, and large with indices of 0.15, 0.25, and 0.35, respectively. The interpretation of effect size is based on Erceg-Hurn and Mirosevich’s (2008) proposal, where 0.2, 0.5, and 0.8 are interpreted as small, medium, and large effect sizes, respectively. To analyze the data, the statistical software JASP Team (2020) version 0.13.1 was used.

## Results

We will present the results starting by showing the sociodemographic variables, then the correlations between all the study variables, and finally, the analysis of membership and social identification of the neighborhood in relation to WB and health.

### Sociodemographic Differences

Concerning the urban–rural origin, only differences are observed in NSI (*t* = −6.95, *p* < 0.001, and *d* = −0.45) and perceived loneliness (*t* = −4.07, *p* < 0.001, and *d* = −0.26), with both scores being lower in the rural population than in the urban one. We do not find differences between urban and rural groups in other variables (health, economic security, life satisfaction, mental–emotional WB, and positive or negative emotions).

Regarding the educational level of the elderly, we found differences in the NSI [*F*(3, 1,465) = 4.60; *p* = 0.001; and *η*^2^ = 0.009] and membership [*F*(3, 1,459) = 3.21; *p* = 0.02; and *η*^2^ = 0.006], and in both cases, these differences had minimal effects. In the case of the NSI variable, differences are found between the groups with primary education and completed secondary education levels [mean difference = 0.12; *t*_(1189)_ = 2.85, *p*_tukey_ = 0.02], where the former group has more NSI than the latter group. In the case of the membership variable, differences are found between the groups with higher and lower educational levels (mean difference = −0.23; *t*_(990)_ = −2.72, and *p*_tukey_ = 0.03), where the former group participates in fewer groups compared with the latter group.

In relation to sex, differences are observed in health (*t* = −4.60, *p* < 0.001, and *d* = −0.24) and emotional–mental WB (*t* = −2.26, *p* = 0.02, and *d* = −0.12), both of which are lower for women than for men. In the case of negative emotions (*t* = 5.23, *p* < 0.001, and *d* = 0.28), women obtained higher scores than men (*t* = −5.23, *p* < 0.001, and *d* = 0.28). No statistically significant differences were found in NSI, economic security, life satisfaction, positive emotions, or perceived loneliness.

### Global Correlational Analysis

A general overview of correlations for the total study variables is provided in Table 1. It is observed that membership correlates negatively with NSI, perceived economic security, mental–emotional WB, and positive emotions. NSI correlates positively with health, perceived economic security, life satisfaction, mental–emotional WB, and positive emotions. It is observed that the NSI variable significantly impacts all the WB indicators, while the membership variable is related to only one of them (positive emotions).

**TABLE 1:** Pearson’s correlation between social identification, health, and hedonic well-being.

	Social identification		Health	Hedonic well-being				
	1	2	3	4	5	6	7	8
1. Membership	–							
2. NSI	−0.19***	–						
3. Health	−0.05	0.18***	–					
4. LS	−0.05	0.18***	0.35***	–				
5. MEWB	−0.07**	0.23***	0.42***	0.46***	–			
6. PE	−0.14***	0.25***	0.40***	0.43***	0.50***	–		
7. NE	0.05*	−0.19***	−0.48***	−0.35***	−0.44***	−0.45***	–	
8. PL	0.04	−0.07**	−0.23***	−0.25***	−0.33***	−0.31***	0.31***	
*M*	3.33	3.52	2.59	7.28	5.39	4.01	2.72	1.45
*SD*	0.86	0.63	0.89	2.01	1.32	0.72	0.77	0.59
*sk*	−1.02	−0.26	0.49	−0.51	−0.98	−0.46	0.08	1.31
*k*	0.34	0.62	0.56	−0.05	0.49	−0.32	−0.10	0.80

Social identification: Membership, membership organization to belonging; NSI, neighborhood social identification. Health, health perception. Hedonic well-being: LS, life satisfaction; MEWB, mental–emotional well-being; PE, positive emotions; NE, negative emotions; and PL, perceived loneliness.*p < 0.05, **p < 0.01, and ***p < 0.001.

From the WB indicators’ point of view, health is positively related to mental–emotional WB, to the absence of negative emotions, and to the existence of positive emotions and to life satisfaction and perceived economic security. Perceived economic security had a large correlation with all positive WB indicators (life satisfaction, mental–emotional WB, and positive emotions).

The membership variable correlates with indicators of WB and health differently by sex. For women, it correlates statistically significantly and negatively with health (*r* = −0.09, *p* < 0.01), perceived economic security (*r* = −0.10, *p* < 0.01), life satisfaction (*r* = −0.07, *p* < 0.05), mental–emotional WB (*r* = −0.09, *p* < 0.05), and positive emotions (*r* = −0.14, *p* < 0.001) and positively with negative emotions (*r* = 0.08, *p* < 0.05). In the case of men, the membership variable only correlates with positive emotions (*r* = −0.16, *p* < 0.001).

### Membership and Neighborhood Social Identification Analysis

The membership variable distribution analysis shows that most of the sample participants do not participate in any of the groups considered (52.07%). The percentage that follows in second place corresponds to participants who are members of only one organization (30.51%), then to a group that participates in two organizations (12.14%) and, finally, a small group of people who belong to three organizations (4.47%); 0.81% of the participants did not answer this question.

Association analysis between high and low membership and high and low NSI on WB and health is found in Table 2. People in the high-membership group show only one difference compared with the low-membership group: it is in the variable positive emotions. In contrast, the high NSI group obtained higher scores in all the WB variables analyzed (health, life satisfaction, mental–emotional WB, and positive emotion) than participants of the low NSI group. Additionally, the group with high social identification with the neighborhood also obtained significantly lower scores in negative emotions and loneliness than the group with low social identification with the neighborhood.

**TABLE 2:** Comparison of the average of health and well-being in older adults according to their level of high or low membership and neighborhood social identification.

	Membership									Neighborhood social identification								
	High			Low						High			Low					
	*M*	*SD*	*N*	*M*	*SD*	*N*	*t*	*p*	*d*	*M*	*SD*	*N*	*M*	*SD*	*N*	*t*	*p*	*d*
Health	2.67	0.79	66	2.54	0.90	766	1.13	0.26	0.15	2.69	0.90	696	2.49	0.87	769	4.26	<0.001	0.22
LS	7.30	1.61	66	7.17	2.06	759	0.52*	0.61	0.07	7.53	1.84	695	7.05	2.13	763	4.60*	<0.001	0.24
MEWB	5.40	1.32	65	5.28	1.31	761	0.68	0.49	0.09	5.64	1.21	696	5.17	1.37	762	6.96*	<0.001	0.36
PE	4.21	0.61	66	3.91	0.74	765	3.23*	0.001	0.41	4.18	0.68	695	3.86	0.72	768	8.60	<0.001	0.45
NE	2.74	0.72	66	2.76	0.79	765	−0.25	0.80	−0.03	2.59	0.80	696	2.83	0.73	768	−5.92*	<0.001	−0.31
PL	1.49	0.67	65	1.47	0.61	763	0.17	0.87	0.02	1.39	0.54	692	1.49	0.62	763	−3.26*	0.001	−0.17

Health, health perception. Hedonic well-being: LS, life satisfaction; MEWB, mental–emotional well-being; PE, positive emotions; NE, negative emotions; and PL, perceived loneliness.*Levene’s test is significant (p < 0.05), suggesting a violation of the equal variance assumption.

To deepen the analysis regarding belonging to organizations, when comparing by membership to the seniors club, it is observed that those who participate in them obtain higher scores in mental–emotional WB (*t* = 1.93, *p* = 0.05, and *d* = 0.12) and positive emotions (*t* = 3.71, *p* < 0.001, and *d* = 0.24) than those older adults who do not report membership in them. In turn, those who reported membership in religious groups presented higher scores in perceived economic security (*t* = 2.57, *p* < 0.001, and *d* = 0.18) than those who did not report membership in religious groups. Regarding neighborhood membership, we compared WB indicators among those who report membership in neighborhood councils with those who do not (Table 3). When comparing the means in the different WB indicators between those who belong and do not belong to the neighborhood council, differences in favor of the former are observed in all the WB indicators. Thus, in general, they present more WB and better health and less negative emotions and perceived loneliness. When we observed the effect of these three different memberships on health and hedonic WB indicators, membership in neighborhood councils has a more significant association with WB than membership in extra-neighborhood groups.

**TABLE 3:** Comparison of means between older adults who are and are not members of neighborhood councils on hedonic health and well-being indicators.

	Yes			No					
	*M*	*SD*	*N*	*M*	*SD*	*N*	*t*	*p*	*d*
Health perception*	2.67	0.82	368	2.56	0.91	1,094	2.21	0.03	0.13
Life satisfaction	7.51	1.88	369	7.21	2.04	1,086	2.55	0.01	0.15
Mental–emotional well-being*	5.56	1.25	369	5.33	1.34	1,086	2.80	<0.001	0.17
Positive emotions	4.14	0.67	367	3.97	0.73	1,092	4.09	<0.001	0.25
Negative emotions	2.64	0.70	369	2.74	0.79	1,092	−2.03	0.04	−0.12
Perceived loneliness*	1.38	0.54	366	1.47	0.60	1,090	−2.66	<0.001	−0.16

*Levene’s test is significant (p < 0.05), suggesting a violation of the equal variance assumption.

**FIGURE 1:**
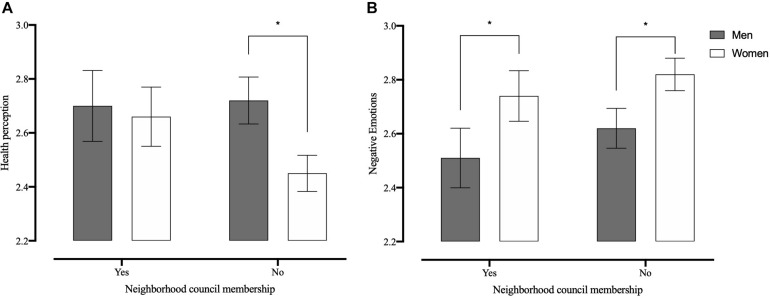
Comparison of perceived health **(A)** and negative emotions **(B)** scores in older adults by sex and neighborhood council membership. *Differences between groups are statistically significant (*p* < 0.05).

Membership in neighborhood councils can be considered a variable whose effect interacts with other variables such as gender. Thus, evaluation of the interaction between belonging to neighborhood councils and gender is presented below to explain its impact on health. A comparison was made to evaluate the impact of sex and belonging to neighborhood councils (nearby territorial organizations) to explain health perception. The results of the 2 × 2 ANOVA for health perception show a main effect for sex, [*F*(1, 1,458) = 8.06; *p* = 0.0005; and *η*^2^ = 0.006], but no main effect for neighborhood council membership [*F*(1, 1,458) = 3.01; *p* = 0.08; and *η*^2^ = 0.003]. However, an interaction effect between both variables [*F*(1, 1,458) = 4.40; *p* = 0.04; and *η*^2^ = 0.003] was found (Figure 1). When examining the simple main effects, there is a higher score in health perception (better health) in men than in women who do not belong to neighborhood councils (*F* = 23.92; *p* < 0.001), which is not observed among those who are members of the neighborhood council (*F* = 0.18; *p* = 0.67).

The same analysis was performed for all WB indicators. The results of the 2 × 2 ANOVA for satisfaction with life show a main effect for neighborhood council membership [*F*(1, 1,451) = 5.55; *p* = 0.02; and *η*^2^ = 0.004], but no main effect for sex [*F*(1, 1,451) = 0.54; *p* = 0.46] or interaction effect [*F*(1, 1,451) = 0.70; *p* = 0.40]. The results of the 2 × 2 ANOVA for mental–emotional WB show a main effect for neighborhood council membership [*F*(1, 1,451) = 6.63; *p* = 0.01; and *η*^2^ = 0.004], but no main effect for sex [*F*(1, 1,451) = 2.18; *p* = 0.14] or interaction effect [*F*(1, 1,451) = 0.62; *p* = 0.43]. The results of the 2 × 2 ANOVA for positive emotions show a main effect for neighborhood council membership [*F*(1, 1,455) = 17.74; *p* < 0.001; and *η*^2^ = 0.01], but no main effect for sex [*F*(1, 1,455) = 1.73; *p* = 0.19] or interaction effect [*F*(1, 1,455) = 1.33; *p* = 0.25]. The results of the 2 × 2 ANOVA for negative emotions show a main effect for sex [*F*(1, 1,455) = 20.40; *p* < 0.001; and *η*^2^ = 0.01] and neighborhood council membership [*F*(1, 1,457) = 3.91; *p* = 0.05; and *η*^2^ = 0.003], but not an interaction effect [*F*(1, 1,457) = 0.05; *p* = 0.82]. The results of the 2 × 2 ANOVA for perceived loneliness show a main effect for neighborhood council membership [*F*(1, 1,452) = 5.67; *p* = 0.02; and *η*^2^ = 0.004], but no main effect for sex [*F*(1, 1,452) = 0.003; *p* = 0.95] or interaction effect [*F*(1, 1,452) = 1.48; *p* = 0.22].

## Discussion

This study identifies the relationships between belonging to the neighborhood and extra-neighborhood social groups, NSI, and their impacts on hedonic WB and health in urban and rural older adults, sexes, and different educational levels. The general results and those related to the hypothesis of the study allow us to draw some conclusions and discuss them with previous literature.

First, it is found that majority of older adults (52%) do not belong to social groups. This result means that participation can be encouraged from public policy with a great possibility that older adults join existing groups, with consequent benefits.

From a theoretical point of view, the two central variables of the social identity approach used here are verified as relevant in their importance to influence health and WB, although they have different impacts. Thus, the two main variables analyzed, membership in local or extra-neighborhood groups and NSI, both have a different relationship with hedonic WB and health. Social membership is not related to life satisfaction nor health. NSI is positively associated with satisfaction with life and health. In fact, the result obtained in this sample of elderly participants indicates that social membership is associated negatively with mental–emotional WB and positive emotions. Of these two, it was social identification with the neighborhood, rather than group membership, which had the most widespread impact on all numbers of WB indicators and with greater intensity as well. Thus, the elderly having a greater NSI (favorable conditions) reported a greater hedonic WB and better health. At the same time, these social identification variables were consistently associated with indicators of hedonic WB.

Regarding the urban–rural differences, the rural older adults presented higher scores in NSI and negative emotions than urban older adults, but not in active social participation. Regarding satisfaction with life, no differences were observed between the urban and rural Chilean samples, which differs from other studies, such as Alvarado et al. (2016) for life satisfaction, or Li et al. (2015), in Chinese samples where rural people had lower levels of happiness and life satisfaction than urban people. The fact that the rural environment in Chile has undergone a process of rapid and growing transformation (urbanization or “new rurality”) perhaps tends to blur the limits and differences with the urban environment, which could explain this result. This has been exacerbated by the accelerated globalization process reaffirmed in 2000 with the signing of the free trade agreement with the United States of America. The inhabitants of urban areas know and expect that the products produced on their lands are exported so their gaze is becoming increasingly global or international. Thus, in rural areas, there is increasing availability of basic goods and services (drinking water, electricity, schools, clinics, pharmacies, cable TVs, and the internet, and among others), which previously defined the identity of urban areas or the urban. This has expanded consumption patterns and transformed rural actors’ mentalities and lifestyles (Canales Echeverría, 2006).

Regarding membership in nearby (neighborhood) and distant groups or organizations (extra-neighborhood), the results are interesting in the sense that the closer they were, the greater the difference was in favor of hedonic WB. Thus, participation in senior citizen clubs or religious groups was not as effective in their impact on WB and health as participation in neighborhood councils. This may be because the neighborhood councils were local (close), territorially delimited on a smaller scale than the clubs for the elderly, which in most cases bring together participants from different neighborhoods of the city. The first facilitates the closest relationships or reinforces those existing in the neighborhood and, thus, social identification. However, both types of participation, in local neighborhood councils or extra-neighborhood groups (religious groups and clubs for the elderly), contribute to WB, giving overall support to the active aging approach. This is in line with the result reported by Sánchez et al. (2017), showing that Chilean older adults trust more in social-civic organizations that are more tightly linked to neighborhoods compared with more distant ones such as political parties (or large private banks). In this way, we believe that this result advances concerning the limited knowledge regarding the activity patterns of older adults (Tomioka et al., 2016; Dawson-Townsend, 2019). Additionally, perhaps the fact of not differentiating between belonging to organizations closer or farther away from home in previous studies explains the contradictory results on WB concerning social participation previously reported in Chile (Alvarado et al., 2016).

The differences between the health perception of men and women coincide with previous literature. Thus, in the sample studied, women presented lower levels of health perception and mental–emotional WB and, in turn, higher scores in negative emotions and frequency of negative feelings. Although these differences appear to be stable according to the literature, it is necessary to consider other variables, such as participation in neighborhood councils, to moderate these results. On the other hand, in life satisfaction, and unlike Alvarado et al. (2016), no differences were observed between sexes.

Social participation in the neighborhood was associated with fewer depression-related elements (perceived loneliness and negative emotions) and higher hedonic WB (mental–emotional WB and positive emotions). It is also associated with a higher health perception. In the case of participation in religious organizations, it was only associated with less perceived loneliness. Thus, our results coincide with previous literature (Chiao et al., 2011; Cruwys et al., 2013; Roh et al., 2015), supporting the idea that participation in heterogeneous groups, despite differences in beliefs and ages but with a shared sense of belonging (neighborhood), is associated with higher levels of hedonic WB and a lower risk of depression.

Finally, when analyzing the effects on health perception, belonging as a neighborhood council member had a significant effect mainly for women. Thus, the health perception of women who participated in neighborhood councils was equal to that of men. These results converge with those of the previous literature regarding the differential impact of social participation on mental health by gender (Kim, 2009; Amagasa et al., 2017), alleviating depressive symptoms (Chiao et al., 2011; Cruwys et al., 2013) and preventing clinical deterioration (Tomioka et al., 2018) in women. In very general terms, social participation in councils favors health. As has been reported, participation is beneficial for maintaining physical function and mental health (Holt-Lunstad et al., 2017). Today, however, given the particular SARS-CoV-2 pandemic situation, social participation in face-to-face groups can put older adults at risk of contagion and, therefore, turn negative.

Finally, from a public policy perspective, our results suggest promoting voluntary membership in social groups, particularly in neighborhood associations, which are ancient forms of organization in the country, and also strengthening urban community actions, which—although focused—will strengthen social identity and belonging to the neighborhood and its groups, which is associated with satisfaction with the place and in line with the social identity approach (Haslam et al., 2009, 2018).

Among the limitations of the study, the following can be mentioned: Firstly, the fact that a pre-existing database was analyzed constitutes a limitation to the type of hypothesis to be verified and the type of measurement instruments used. These are not necessarily the most widely used or conventional instruments on the subject, but they are prepared and applied to fulfill the purposes of a national survey whose purpose is primarily a social diagnosis. Secondly, being a pre-existing survey, not all the hypotheses could be verified as one could wish, but only those possible ones based on the existing database.

## Data Availability Statement

Publicly available datasets were analyzed in this study. This data can be found here: http://epi.minsal.cl/condiciones-de-uso/.

## Ethics Statement

Ethical review and approval was not required for the study on human participants in accordance with the local legislation and institutional requirements. The patients/participants provided their written informed consent to participate in this study.

## Author Contributions

EM-D: responsible for the conception and design of the study. Review of the literature and writing of the work. Support for the analysis or interpretation of data for work. RM-L: responsible for the statistical analysis, interpretation, and description of the results. Support in the revision of different versions of the manuscript, up to the version presented. Both authors contributed to the article and approved the submitted version.

## Conflict of Interest

The authors declare that the research was conducted in the absence of any commercial or financial relationships that could be construed as a potential conflict of interest.

## Footnotes

1 This research used information from the Health Surveys for epidemiological surveillance of the Under Secretariat of Public Health. The authors thank the Ministry of Health of Chile for allowing them to have the database. All the results obtained from the study or investigation are of exclusive responsibility of the authors and in no way involves the abovementioned institution.

## Appendix 1

### Neighborhood Social Identification Scale

1. In general, I feel very comfortable living in this neighborhood.
2. I feel like I belong to this neighborhood.
3. I visit my neighbors at their houses.
4. The friendships and relationships I have with other people in my neighborhood mean a lot to me.
5. If I had the opportunity, I would like to leave this neighborhood (reverse encoding).
6. If people in my neighborhood plan something, I would think that it is something that we are doing together and not something that others are doing.
7. If I need advice, I can ask someone in my neighborhood.
8. I think I agree with most of the people in my neighborhood about what life is important.
9. I think my neighbors could help me in case of an emergency.
10. I feel loyalty to the people in my neighborhood.
11. With my neighbors, we lend each other things and do each other favors.
12. I am willing to collaborate with other people to improve my neighborhood.
13. I plan to continue living in this neighborhood for several years.
14. I like to feel similar to the people who live in my neighborhood.
15. My neighbors rarely visit my home (reverse encoding).
16. I have a feeling of deep fellowship with other people in this neighborhood.
17. I regularly stop to chat with people in my neighborhood.
18. Living in this neighborhood gives me a sense of community.
19. If you dropped a purse or wallet in your neighborhood, on your street, or in your village or town, and someone saw it, do you think he or she would return it to you?
